# Rate-and-state friction explains glacier surge propagation

**DOI:** 10.1038/s41467-019-10506-4

**Published:** 2019-06-27

**Authors:** Kjetil Thøgersen, Adrien Gilbert, Thomas Vikhamar Schuler, Anders Malthe-Sørenssen

**Affiliations:** 1 Physics of Geological Processes, The NJORD Centre, University of Oslo, 0316 Oslo, Norway; 2 Department of Geosciences, University of Oslo, 0316 Oslo, Norway; 3 Department of Physics, University of Oslo, 0316 Oslo, Norway

**Keywords:** Cryospheric science, Geodynamics

## Abstract

The incomplete understanding of glacier dynamics is a major source of uncertainty in assessments of sea-level rise from land-based ice. Through increased ice discharge into the oceans, accelerating glacier flow has the potential to considerably enhance expected sea-level change, well ahead of scenarios considered by the IPCC. Central in our incomplete understanding is the motion at the glacier bed, responsible for flow transients and instabilities involving switches from slow to fast flow. We introduce a rate-and-state framework for the transient evolution of basal shear stress, which we incorporate in glacier simulations. We demonstrate that a velocity-strengthening-weakening transition combined with a characteristic length scale for the opening of subglacial cavities is sufficient to reproduce several previously unexplained features of glacier surges. The rate-and-state framework opens for new ways to analyze, understand and predict transient glacier dynamics as well as to assess the stability of glaciers and ice caps.

## Abstract (web summary)

Predicting the friction at the ice/bedrock interface prevents a full understanding of glacier dynamics. Here, the authors present a framework for the transient evolution of basal shear stress and incorporate it in glacier simulations. This tool is capable of explaining surge onset and propagation.

## Introduction

Understanding transient and fast dynamics of glacier flow is of crucial importance, which is highlighted in the latest IPCC report that states that changes in ice sheets could occur even more rapidly than previously recognized^1^. Our understanding of basal friction remains one of the largest contributors to uncertainties in estimates of future sea-level rise^2^, and while it is widely acknowledged that glacier-flow instabilities are governed by basal processes, several features of these events remain to be captured in models.

Parameterizations of glacier bed friction often assume that the basal shear stress is a strictly increasing function of sliding velocity, and that changes in basal shear stress are instantaneous with changes in stress conditions and sliding velocity. However, glaciers are subject to a wide range of transient phenomena where these monotonic and steady state assumptions are questionable: on short time-scales, stick slip at glacier beds has been detected by induced seismicity^3,4^, on intermediate time-scales, glaciers are subject to transient response to rainfall^5,6^, and glaciers are subject to seasonal variations^7^. The perhaps most striking example of transient glacier dynamics is the onset and propagation of glacier surges, where ice flow velocities can increase by orders of magnitude for a period of time. In extreme cases, glacier surges have even been shown to act as precursors to catastrophic glacier collapse^8^. In such cases it is crucial to understand what controls rapid transitions between slow and fast flow. It is therefore essential to develop improved parameterizations for basal shear stress that reproduce slow, fast as well as transient dynamics of glaciers.

**Fig. 1:**
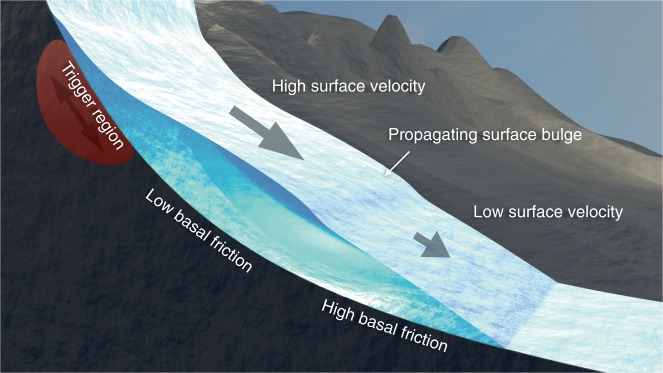
Sketch of the model system based on the Variegated Glacier. A glacier surge often propagates down-glacier from a triggering region accompanied by a traveling surface bulge. To model surge propagation, we introduce a rate-and-state type sliding law that exhibits a transition from velocity-strengthening to velocity-weakening friction where the frictional strength evolves over a characteristic length scale *d*_c_. Low basal friction behind a surge front occurs spontaneously in the model due to this transition, and the propagation of the surge is governed by *d*_c_

Glacier surges usually occur on quasi-regular intervals^9^, and the common conception involves at least two mechanisms behind these instabilities; the thermal and the hydrologic switch mechanism, or a combination thereof^10–18^. Further, the role of driving stress in surge triggering became apparent in the Karakoram, where the frequency of glacier surges increased after a period of glacier mass gain^19,20^. Glacier surges often originate in a small region of the glacier before they propagate both up- and down-glacier. The front of such expanding surge region is recognized as a transition from low to high surface velocities^13,14,21–23^, and the down-glacier propagation of this front manifests as a characteristic traveling surface bulge^13^ (Fig. 1). Propagation speeds typically reach kilometers per year, and inversion of basal characteristics during a surge has revealed that basal shear stress evolves with time and decays behind the surge front^24^.

Several features of glacier surges remain unexplained; in particular how and whether a local perturbation grows into a glacier-wide surge, as well as the relations between sliding velocity, propagation speed, and ice thickness change. While the relation between propagation speed, sliding velocity, and elevation change can be understood from volume conservation arguments^14^, the mechanism controlling the relation between flow velocity and front speed during glacier surges remains elusive. The relation between basal shear stress, sliding velocity, and water pressure is vital for understanding glacier surges^12^. Several models have been able to produce cyclic flow velocities from feedback mechanisms between strain heating and water pressure^16,17,25,26^. It has also been suggested that flow instabilities can occur as a result of velocity-weakening friction at the glacier bed. This idea was introduced by Lliboutry in 1964^27^, and since then several authors have discussed the role of multi-valued sliding laws for unstable glacier flow, both in terms of velocity-weakening friction^28,29^, and in terms of fast and slow branches of sliding arising from a switch between distributed and channelized drainage^30,31^. Even though these pioneering ideas have been well known for a long time, few attempts exist in using these concepts to understand the propagation of glacier-flow instabilites.

In 1981, Iken suggested that there is an upper bound on the shear stress that a hard-bed glacier bed can support^32^ due to opening of subglacial cavities, which has been shown to exist for arbitrary bed geometries as long as the slopes are bounded^33^. This can lead to a transition from velocity-strengthening to velocity-weakening friction above transitional sliding velocity. A similar transition can also occur for due to particle ploughing in subglacial tills^34^. Recent theoretical approaches build on the ideas on cavitation at hard-bed glaciers^33,35^. Gagliardini et al. performed finite element simulations of ice sliding over rigid bedrock under temperate conditions. From these simulations they proposed a parameterized sliding law that relates basal shear stress to velocity and effective normal stress^35^. This relation is now widely used in glacier simulations. The basal shear stress is velocity-strengthening at low velocities, before it reaches a maximum threshold and becomes velocity-weakening. Non-monotonic basal shear stress for hard-bed glaciers has also been observed experimentally^36^. Even though it is well known that glacier beds can exhibit velocity-weakening basal shear stress, the models for basal shear stress used in numerical simulations of glaciers are often assumed to be strictly increasing with velocity^37^. The parameterization by Gagliardini et al. is also usually applied in a monotonic configuration (*q* = 1 in Eq. (2)).

Recent experiments have revealed that the frictional response of ice sliding over a hard bed is not instantaneous^36,38–40^. This includes temperature dependent interfacial healing^40^ and non-instantaneous opening of subglacial cavities^36^. Such effects are yet to be fully incorporated in parameterizations of basal shear stress, which usually assume that changes in basal shear stress are instantaneous with changes in stress conditions and velocity.

In the following, we introduce a novel rate-and-state formulation for the transient evolution of basal shear stress for glaciers sliding over rigid bedrock. Through numerical simulations, we demonstrate that a transition from velocity-strengthening to velocity-weakening friction combined with a characteristic length scale for the evolution of subglacial cavities is sufficient to capture the onset and propagation of glacier surges, including a traveling surface bulge, the relation between ice velocity and propagation velocity as well as the time-dependent evolution of basal shear stress. The results are compared with observations from the Variegated Glacier surge of 1982.

## Results

### Basal shear stress and glacier stability

We introduce a delayed frictional response due to non-instantaneous opening of subglacial cavities over a characteristic length scale *d*_c_ by extending the model of Gagliardini et al.^35^. For a given ice geometry at the interface, Weertman’s relation for basal shear stress as a function of velocity applies, but is modified by a dimensionless state parameter *θ* that describes the degree of cavitation and takes values between 0 and 1

1 $$\tau _{\mathrm{b}} = \theta \left( {\frac{v}{{A_{\mathrm{s}}}}} \right)^{1/m}.$$

The functional form of Eq. (1) has also been demonstrated experimentally^39^. In steady state, at the pressure melting point, the basal shear stress is given by the parameterization by Gagliardini et al.^35^

2 $$\tau _{{\mathrm{b}},{\mathrm{ss}}} = \sigma _{\mathrm{N}}C\left( {\frac{\chi }{{1 + \alpha \chi ^q}}} \right)^{1/m},$$

where $\chi = \frac{v}{{C^m\sigma _{\mathrm{N}}^mA_{\mathrm{s}}}}$, and $\alpha = \frac{{(q \, - \, 1)^{q - 1}}}{{q^q}}$, and *σ*_N_ is the effective normal stress. The friction law contains three geometrical parameters, *C*, *A*_s_, and *q* with the following physical interpretation: *Cσ*_N_ is the maximum shear stress the bed can support, and *C* is set by the maximum slope of the bed roughness. *A*_s_ relates the sliding velocity and the basal shear stress in the absence of cavitation. *q* determines the post-peak decay of the friction law, and is an increasing function of the ratio between the maximum and the mean slope of the bed roughness^35^. The state parameter in steady state can be found by combining Eqs (1) and (2) and is given by $\theta ^\dagger = \left( {\frac{1}{{1 + \alpha \chi ^q}}} \right)^{1/m}$. If the cavity opening is governed by a characteristic length scale *d*_c_ and the evolution toward steady state is exponential, the state evolution law is given by

3 $$\frac{{\partial \theta }}{{\partial t}} = \frac{v}{{d_{\mathrm{c}}}}(\theta ^\dagger - \theta ).$$

**Fig. 2:**
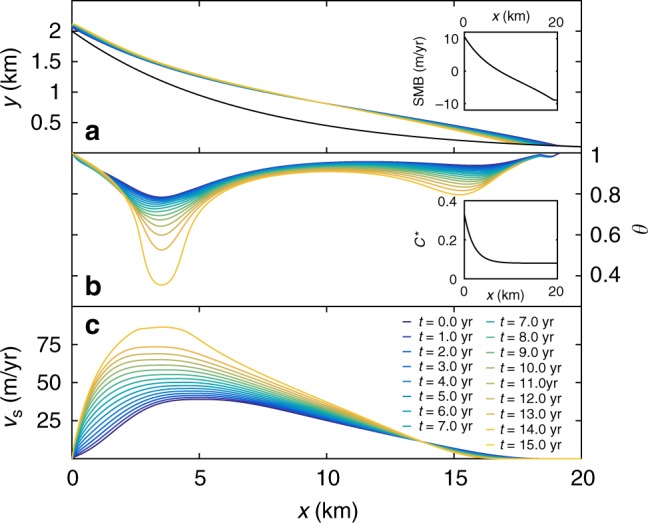
Evolution of topography, basal friction and surface velocities during the quiescent phase. **a** Evolution of the glacier geometry in time. Inset: the prescribed surface mass balance (SMB) is a function of elevation, here plotted as a function of *x* using the initial glacier geometry. **b** Evolution of the state parameter *θ*. Inset: the peak friction coefficient $C^ \ast = C\frac{{\sigma _{\mathrm{N}}}}{{\sigma _n}}$ (details in Supplementary Information) as a function of *x*. **c** Evolution of surface velocity. Simulation using *q* = 1.5 and *d*_c_ = 1.5 m

The interpretation of *d*_c_ as a characteristic length scale for cavity development implies that its value should be in the meter range, which is also consistent with the experimental observations by Zoet et al.^36^. The response of the sliding law to stepwise changes in velocity is in qualitative agreement with experiments (Fig. 2), and shares several features with classical rate-and-state friction (rate refers to the velocity-dependence of the basal shear stress while the state refers to the current state of cavitation at the glacier base). To account for lateral drag, we include an additional body force in the simulations ${\vec{\mathbf{F}}}_{{\mathrm{lateral}}} = - K|{\vec{\mathbf{v}}}|^{\frac{1}{n} - 1}{\vec{\mathbf{v}}}$, where *K* can be related to the glacier width and *n* is Glen’s exponent^41^.

The transient evolution of subglacial cavities has important consequences. In particular, a stability criterion for the onset of rapid dynamics is an inherent property of the sliding law, which has important implications for surging glaciers; at low driving stresses where the basal shear stress increases with velocity, the glacier will be stable. However, if the basal shear stress locally exceeds *Cσ*_N_, there is a possibility for unstable motion, if the size of the region reaching *Cσ*_N_ is larger than a critical length scale $L_{{\mathrm{critical}}} \simeq - \left( {\frac{v}{{d_{\mathrm{c}}\dot \varepsilon \nu ^ \ast }}\frac{{\partial \tau _{{\mathrm{b,ss}}}}}{{\partial v}}} \right)^{ - 1},$ where $\dot \varepsilon \nu ^ \star$ is the viscous stress in a patch of size *L*_critical_ in the velocity-weakening regime (details in Supplementary Information).

### Surge trigger and propagation

**Fig. 3:**
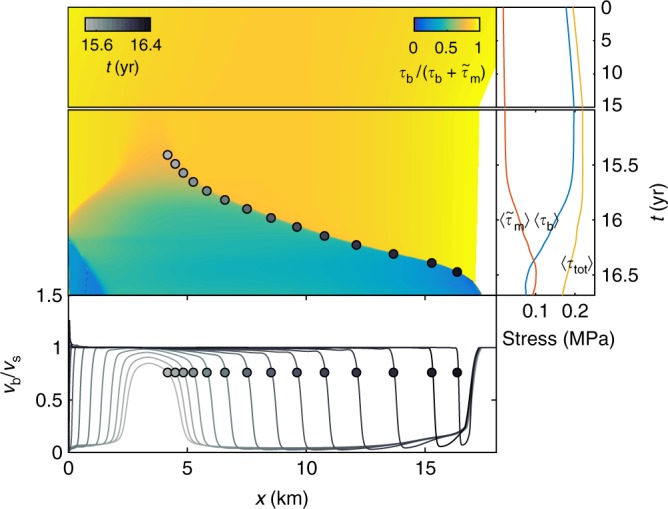
Stress is transferred from the base to the margins during the surge. The velocity at the base becomes approximately equal to the velocity at the surface behind the surge front. The main panel shows the fraction of spatiotemporal shear stress *τ*_b_, supported by the base. $\tilde \tau _{\mathrm{m}}$ is the shear stress from the margins calculated by Supplementary equation 16. The right panel shows the average basal shear stress 〈*τ*_b_〉, margin stress $\langle \tilde \tau _{\mathrm{m}}\rangle$ and the average driving stress 〈*τ*_tot_〉 as a function of time. There is a gradual build-up of stress before the surge starts around *t* = 15.5 years. During the surge, stress is transferred from the base to the margins, and the driving stress decreases. The bottom panel shows the ratio of basal and surface velocity for different instances in time during the surge. The grayscale dots correspond to the grayscale dots in the main panel and are uniformly spaced in time with intervals of 30 days. Behind the surge front, the basal and surface velocities are more or less equal. The simulation in the figure used *d*_c_ = 1.0 m, *q* = 1.5

We consider an instability triggered through a change in the ratio *τ*/*σ*_N_ due to changes in either ice thickness or meltwater supply. We use an idealized geometry of the Variegated Glacier (Supplementary Figure 1), with initial situation shown in Fig. 2. The evolution of ice thickness, frictional strength and the resulting surface velocities in the 15 years before surge onset are shown in Fig. 2. The time before surge onset is dependent on the choice of *q* (from approximately 12 to 15 years between *q* = 2.0 and *q* = 1.5 with parameter set 1 in Supplementary Table 1), which is related to both the slight change in basal sliding velocities close to the velocity-weakening transition and the dependence of the stability criterion on *q*. For *q* below 1.5, no surge is triggered within 20 years of simulation time. In the quiescent phase, the driving stress gradually increases due to the change of surface mass balance (Fig. 3). The frictional properties at the base develop continuously due to the changes in topography affecting the normal and shear stresses at the base, as is visible by the significant decrease of *θ* (Fig. 2). In our sliding law, this is interpreted as an increased degree of cavitation. There is a small region around *x* = 3 km that experiences the largest change.

**Fig. 4:**
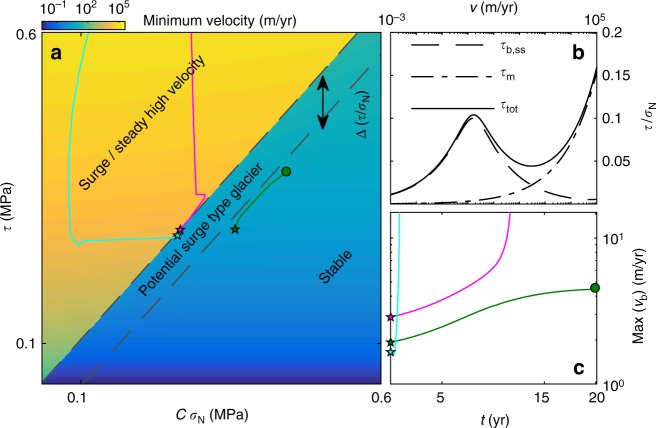
Combined basal and lateral drag is velocity-strengthening at low velocities, velocity-weakening at intermediate velocities, and velocity-strengthening at high velocities. The smallest possible sliding velocity that can support a driving stress *τ* for a given *Cσ*_N_ (**a**) derived from the steady state sliding law including stress from the glacier margins (**b**) given C* = 0.1, and the driving stress for *A*_s_ = 100 m yr^−1^ MPa^−3^, *n* = 3, *m* = 3, *A* = 15 yr^−1^ MPa^−3^, *W* = 1.5 km, *q* = 2.0 and *σ*_n_ = 3.5 MPa (Supplementary equation 15). *τ*_b,ss_ is the basal shear stress, *τ*_m_ is the stress from the margins, and *τ*_tot_ is the total shear stress. The steady state velocity in **a** demonstrates a discontinuity from slow sliding regime dominated by basal friction to a fast sliding regime where shear stress is carried at the glacier margins. Surges can be triggered if the line of stability (black dashed line) is crossed in a sufficiently large region of the glacier bed given by *L*_critical_ (details in the text). Colored lines show the driving stress *τ* and *Cσ*_N_ at the position at the base with the largest value of *τ* (excluding the up-glacier propagation). **c** The maximum basal velocity for the simulations shown in **a**. All simulations were performed with *q* = 2.0 and *d*_c_ = 1.0 m, but with different values of C*. Magenta: C* = 0.25e^*x*/2000m^ + 0.08 (System studied in main text). Green: C* = 0.25e^*x*/2000m^ + 0.12, Cyan: *C* = 0.3 with a full hydrology model triggered by an increase in meltwater supply at the base (details in Supplementary Information methods and Supplementary Figure 8 and Supplementary Figure 9)

Once the instability criterion is reached locally, the surge starts. A rapid increase in sliding velocity is predicted from the combined basal and lateral drag, which can be approximated as an effective friction coefficient at the base (Fig. 4). The effective friction coefficient is velocity-strengthening at low velocities, velocity-weakening at intermediate velocities, and velocity-strengthening at high velocities when the lateral friction is dominant. The minimum steady state sliding velocity that can support a given driving stress *τ* and effective normal stress *σ*_N_ is discontinuous at *τ* = *Cσ*_N_ because the driving stress is transferred from the base to the margins. Surges can be triggered if a sufficiently large region of the glacier bed reaches *τ*/*σ*_N_ > *C* and enters the velocity-weakening regime. Multiple processes can act as a trigger mechanism; we have triggered surges by changes in both water pressure and changes in mass balance. Note that this formulation can also describe steady fast flow with the additional requirement of a sufficiently large shear stress to stay in the high velocity regime where the lateral margins carry most of the shear stress.

**Fig. 5:**
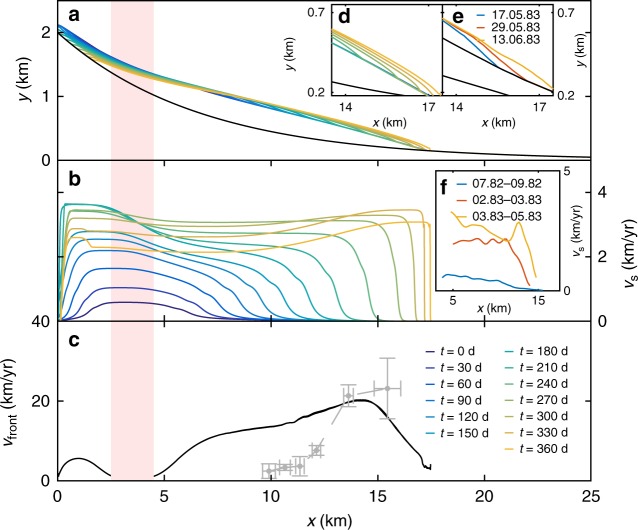
Surge propagation in the model including qualitative comparison with the Variegated Glacier surge. **a** Evolution of the glacier geometry with time during a surge. **d** Detail of the simulated geometry. **e** Surge front of Variegated Glacier adapted from Kamb et al.^13^. **b** Evolution of surface velocities in time. **f** Variegated glacier velocities from Jay-Allemand^24^ (the acceleration at the end of the surge is omitted). **c** Surge propagation speed as a function of front position downstream, dashed line shows surge propagation velocities from the Variegated Glacier. The red marker shows the trigger region. Simulation performed with *d*_c_ = 1.0 m and *q* = 1.5

The initial phase of the surge is visible as a surface bulge that develops downstream of the triggering area, with a corresponding surface depression upstream. Next, the region of increased velocities expands in both directions, accompanied by a traveling surface bulge. Figure 5 shows the evolution of the glacier geometry, surface velocities, and the corresponding surge propagation speed during a surge. For the particular set of parameters, the surge velocities reach 3 km/year, and the surge propagation speed 20 km/year. The thickness of the surface bulge reaches 100 m, and the duration of the surge propagation is roughly 1 year until the entire glacier is affected. Figure 5 also shows observations of the Variegated Glacier surge by Jay-Allemand et al.^24^ to demonstrate the similarities with our simulations. The net effect of the surge is to decrease the glacier driving stress (Fig. 3). During the surge, stress is transferred from the base to the margins so that the increased flow velocities are completely dominated by basal sliding.

**Fig. 6:**
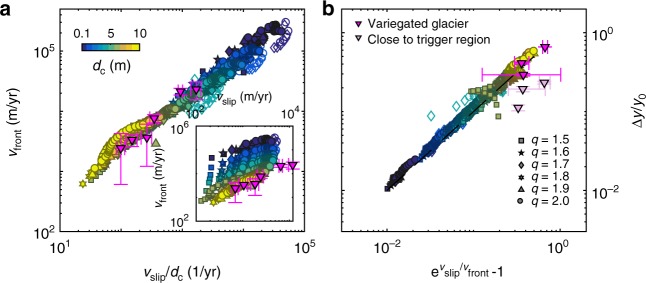
Scaling relations for the propagation velocity, the sliding velocity and the surface elevation change. **a** Dependence of surge front velocity *v*_front_ on sliding velocity *v*_slip_ ∀*x* ∈ [5, 15] km. The scaling relation *v*_front_ ~ *v*_slip_/*d*_c_ applies for simulations as well as observational data from the Variegated glacier (with *d*_c_ = 7 m). **b** Relative elevation change as a function of ${\mathrm{e}}^{v_{{\mathrm{slip}}}/v_{{\mathrm{front}}}} - 1$ ∀*x* ∈ [7.5, 15] km, where elevation change Δ*y* is measured 250 m behind the surge front. The dashed line shows the exact relation. The figure also includes data from the Variegated glacier (Supplementary Figures 3 and 4), where we have used a different color on the points close to the trigger region where we do not expect the relation to be accurate. Open symbols show results using a full hydrology model (parameter set 2 in Supplementary Table 1), while closed symbols show results from the simplified model (parameter set 1 in Supplementary Table 1)

Figure 6 shows the relation between sliding speed, propagation speed, and bulge thickness. The ratio between sliding speed and the front speed is directly controlled by the length scale *d*_c_, and we collapse the data from all simulations, including also data from the Variegated surge where we set *d*_c_ = 7 m, on a single line (scaling relations are given in SI). We find a weak dependence of *q* on both the sliding velocity and the front velocity, but the ratio between the sliding speed and front propagation speed is fairly constant over changes in *q*. The net result is that larger values of *d*_c_ lead to both slower propagation and slightly lower velocities, but speed saturates at low values *d*_c_ when stress is supported by the glacier margins. However, larger values of *d*_c_ also show more transient evolution of the sliding speed during the surge compared with lower values of *d*_c_ (Supplementary Figures 5–7). Lower values of *q* leads to slightly lower velocities. Over the range of investigated parameter values, the sliding speeds and propagation speeds span several orders of magnitude. The surface bulge thickness can be found from volume conservation arguments and is determined by the ratio between sliding and front speed. The surface bulge thickness is not sensitive to changes in *q* because the ratio between the sliding speed and the front speed remains fairly constant. However, due to the direct influence of *d*_c_ on sliding and front speed, the bulge thickness is highly sensitive to the value of *d*_c_. Slow propagation leads to more prominent elevation changes. Observations from Variegated Glacier support this relation.

## Discussion

The sliding we have introduced is mathematically similar to the classical rate-and-state friction laws. However, we stress that the rate-and-state type law we have introduced here is not based on the same physical interpretation as the classical rate-and-state laws. In this study, a state parameter is introduced to capture the transient evolution of subglacial cavities to changes in sliding velocity or effective normal stress, whereas the classical rate-and-state friction law motivates the state parameter from microscopic asperity contacts.

The glacier geometry used here idealizes the Variegated Glacier. The approach taken here has been to focus on the mechanisms behind glacier surges rather than precisely reproducing all aspects of one particular surge. This means that the comparison in Fig. 5 is qualitative. However, we have systematically varied model parameters and developed scaling relations for surge propagation speed and elevation change that we have tested against observations.

Still, we find it useful to briefly discuss similarities and differences between our simulations and the Variegated Glacier surge of 1982. In the 10 years prior to the Variegated surge of 1982, there was a gradual change in ice thickness, as well as a gradual velocity increase including large seasonal variations^42^. Qualitatively, the build-up to the surge is similar in our simulations, but without the seasonal variations. In the beginning a surge bulge formed with height of around 30 m, and consecutively propagated downstream at an average speed of 8.4 km/year in the upper part, reaching velocities of almost 30 km/year in the lower part^13^. The range of velocities obtained from our simulations agrees well with observed surge velocities, and we found that a characteristic length scale of 7 m for the evolution of frictional strength is consistent with observations from the Variegated Glacier. The thickness of the bulge observed on the Variegated Glacier is consistent with our scaling relation that depends on the ratio of the sliding speed and the front speed. During the surge as much as 95% of the motion has been attributed to basal sliding^13^. Our simulations are in good agreement with these observations, and they also show time-dependent evolution of frictional strength that are in qualitative agreement with results from inversion of the frictional properties during the Variegated Glacier surge^24^. Surge speed and duration vary widely in nature^9^. Our simulations demonstrate that sliding and propagation speeds can vary over orders of magnitude depending on the details of the frictional response at the base. Consistently high basal water pressures were found during the Variegated Glacier surge^13^. The role of basal water is also evident from the large discharge of water that coincided with the sudden surge termination. In our simulations we do not reproduce sudden surge termination. This suggests that there should be direct couplings between the subglacial drainage system and the cavity evolution that we do not model explicitly here.

Cavitation is often included in subglacial hydrology models assuming drainage through a cavity sheet (e.g., ref. ^43^). Here, we have considered the effect of such cavitation on the frictional response of the glacier bed. This has important consequences for understanding the triggering of glacier flow instabilities through the introduction of a critical nucleation length. Interestingly, this framework captures both down and up-glacier surge propagation which is often observed^44^. As subglacial cavitation is tightly connected to both friction and drainage, we suggest that future approaches should aim to combine the two effects in a single friction-hydrology framework.

The critical nucleation length should in principle be observable in the initial stages of glacier surges as the size of the region of increased velocity just prior to the onset of propagation. Such observations are scarce, but the improved temporal resolution of satellite data collected over the last few years show great promise. In particular, a recent study by Altena et al.^44^ clearly demonstrates that a surge triggered in a region in the mid part of the Klutlan glacier in 2014 before it started to propagate both up- and down-glacier.

Glacier sliding over hard bed is a limiting assumption. Glacier surges have been found to often occur for glaciers resting on layers of soft, deformable sediments^12^. Even though our rate-and-state sliding law is developed for hard beds, soft beds will exhibit similar surge behavior if the friction law contains a transition from velocity-strengthening to velocity-weakening and accounts for non-instantaneous evolution of frictional strength. Chen et al. have developed a friction framework that bears similarities to rate-and-state friction valid for fault gouges^45^. They demonstrate a transition from velocity-strengthening to velocity-weakening friction at low sliding velocities due to a transition from non-dilatant plastic creep to dilatant granular flow and contact creep. The model predicts that the characteristic length scale for frictional evolution in a granular fault gouge scales as *d*_c_ ~ *Lλ*, where *L* is the thickness of the entire gouge layer and *λ* is degree of localization. If such model can be applied also at glacier beds, the value of *d*_c_ ≃ 7 m found to yield a data-collapse of the relation between *v*_front_ and *v*_slip_ for the Variegated Glacier surge would be consistent with at least two different interpretations; a hard-bed glacier with a characteristic cavity size of 7 m, or a soft bed glacier with a relatively thick sediment layer and a low degree of localization. For the Variegated glacier where sediments have been detected at the bed^46^, this offers a plausible explanation of the observed relation between *v*_front_ and *v*_slip_, although this is not consistent with the experimental observations of strain localization in sheared tills^47^.

In the following, we place our rate-and-state framework in the context of current theories of glacier surge mechanisms. In this paper we have focused on the propagation of glacier instabilities, and we find that surge propagation is governed by reaching a stability criterion and a subsequent transition to a velocity-weakening friction regime. Even though instabilities can be triggered by various mechanisms, the driving factor of the surge propagation within our simulations is not due to feedback mechanisms between strain heating, water pressure, and subglacial drainage (although redistribution of basal water toward the glacier front can cause the surge to speed up as can be seen in Supplementary Figure 9). Such feedback mechanisms have been demonstrated to be the driving factor in models able to produce cyclic flow velocities^16,17,25,26^. In that respect, our rate-and-state framework does not alone provide an explanation for glacier surge behavior, but it does provide a framework for surge propagation compatible with current surge theories. In particular, our rate-and-state framework introduces a stability criterion for the onset of propagation that may explain why certain glaciers have speed up events that do not evolve to glacier-wide surges^12^, and it is capable of explaining the relationship between sliding speed, propagation speed and ice thickness change during surges. It also has the potential to improve our comprehension of ice dynamics by introducing a way to incorporate rate-weakening friction in numerical models. However, we stress that in order to make quantitative predictions future development of our rate-and-state framework should include feedback mechanisms between strain heating, meltwater production, subglacial drainage, and cavity evolution as well as temperature effects^40,48^. Despite the assumptions made in this study, the overall surge behavior within our model is strikingly similar to observations.

We have demonstrated that a rate-and-state friction framework including a velocity-strengthening-weakening transition and a characteristic length scale for the transient evolution of basal shear stress is capable of explaining surge onset and propagation. The local onset of glacier surges, a traveling surface bulge, the relation between ice velocity and propagation velocity, and the time-dependent evolution of frictional strength are all captured within our simulations. Our framework has the potential to improve our understanding of any class of glacier dynamics where transient evolution and velocity-weakening basal shear stress is present. In particular, it provides a new tool to understand glacier surges, ice avalanches, or any switch from slow to fast motion of glaciers and ice caps.

## Methods

### Numerical simulations

To solve for the ice flow we use the open source finite element code Elmer/Ice^49^ and use an idealized version of the Variegated glacier as our glacier geometry (Supplementary Figure 1). We use Glen’s flow law^12^. At the upper and lower boundaries we have zero velocity boundary conditions in the horizontal direction and free slip boundary conditions in the vertical direction. We solve for two different parameter sets given in Supplementary Table 1. First, we assume that the water pressure is assumed to be proportional to the normal stress, which reduces the number of parameters in the system, highlighting the role of velocity-strengthening–weakening friction in the propagation of glacier surges. Surges are triggered by changes in surface mass balance. Second, we include a full hydrology model^50^, and trigger surges by a perturbation in meltwater supply (details in Supplementary Information).

## Supplementary information

Supplementary Information

Source Data

## Data Availability

Run scripts that allow for reproduction of the data set is included in the supplementary materials. In addition, data generated from simulations are available from the authors on reasonable request.

## Code availability

The supplementary materials contains run scripts and source code that combined with Elmer/Ice allows the reader to reproduce the dataset.
