# Supplementary material for: Rate-and-state friction explains glacier surge propagation
Source: Nat Commun. 2019 Jun 27;10:2823. doi: 10.1038/s41467-019-10506-4 (PMC6597537; doi:10.1038/s41467-019-10506-4)
Supplement: Supplementary file 1 — Supplementary Information [file 41467_2019_10506_MOESM1_ESM.pdf]

Supplementary information for: Thøgersen et al., Rate-and-state friction explains glacier surge propagation

SI Materials and Methods

We solve the ice flow using open source finite element code Elmer/Ice [1]. Including lateral friction, conservation of momentum yields

$$\nabla \cdot \underline{\boldsymbol{\sigma}} = -\rho \vec{\mathbf{g}} - K|\vec{\mathbf{v}}|^{1/n-1}\vec{\mathbf{v}}, \tag{1}$$

where ρ is the density, $\vec{\mathbf{g}}$ is the gravitational acceleration and $\underline{\boldsymbol{\sigma}}$ is the Cauchy stress tensor

$$\underline{\boldsymbol{\sigma}} = 2\nu\dot{\underline{\boldsymbol{\epsilon}}} - p\underline{\boldsymbol{I}}. \tag{2}$$

Here, p is the pressure, $\underline{\boldsymbol{I}}$ is the identity matrix, $\dot{\underline{\boldsymbol{\epsilon}}}$ is the strain rate tensor and ν is the effective viscosity

$$\nu = \frac{1}{2}A^{-\frac{1}{n}}\dot{\epsilon}_e^{\frac{1-n}{n}} \tag{3}$$

where $n = 3$ is Glen's exponent, $\dot{\epsilon}_e = \sqrt{\frac{\mathrm{tr}(\dot{\underline{\boldsymbol{\epsilon}}}^2)}{2}}$ and A is the Arrhenius factor. We assume that the ice is incompressible so that conservation of mass is given by

$$\nabla \cdot \vec{\mathbf{v}} = 0. \tag{4}$$

In addition, we solve for the evolution of the free top surface elevation h through

$$\frac{\partial h}{\partial t} + v_x\frac{\partial h}{\partial x} - v_y = SMB, \tag{5}$$

where SMB is the prescribed surface mass balance shown in Supplementary Figure 2.

For systematic studies of surge propagation we use a simplified approach for the basal hydrology. Instead of manipulating both the water pressure and the the peak friction coefficient C, we have modified C directly. This is mathematically equivalent to assuming that the water pressure p_w is proportional to the normal stress σ_n. Even though the value of the maximum basal shear stress C is usually not known, we can set up the system close to the stability threshold by modifying the water pressure. This can be written as $p_w = f\sigma_n$, we have that $\sigma_N = (1-f)\sigma_n$ so that

$$C\sigma_N = C(1-f)\sigma_n \equiv C^*\sigma_n. \tag{6}$$

Here, we choose C^* so that the system is close to the critical threshold, and so that the frictional characteristics are qualitatively similar to the inversion of basal friction by Jay-Allemand et al. [2]; $C^*(x) = 0.25e^{-x/2000m}+0.08$. We further neglect seasonal variations in basal water pressure, so that a surge is triggered by changes in τ_b/σ_N due to topography changes alone. The simulation is stopped when the surge reaches the terminus. The parameters used in the simulations are given in table 1.

We performed systematic simulations with $q \in [1.5, 2.0]$ and $d_c \in [0.1, 10]$m. The systematic runs with changed d_c were restarted from simulations with $d_c = 1$m just after surge onset to reduce computational cost.To measure the front velocity we used $\theta(x,t)$ and traced the treshold value $\theta_{\mathrm{threshold}} = 0.25$. Front velocity could in principle be measured from most of the variables at the glacier bed, but θ exhibits the sharpest transition at the front tip (Supplementary Figure 6 and 7).

We also perform a second set of simulations using the double-continuum hydrology model for subglacial water pressure by de Fleurian et al. [3]. We refer to their paper for a detailed description of the model, but give a brief overview of the model here. The model assumes an inefficient drainage system (IDS) and an efficient equivalent porous layer (EPL). Initially the EPL is inactive. The model solves the vertically integrated Darcy's equations for a confined aquifer for both layers

$$S\frac{\partial h_w}{\partial t} - \nabla \cdot (T\nabla h_w) = Q \tag{7}$$

where Q is the source flux per unit surface, T is the transmissivity, S is the storage coefficient of the porous media, and h_w is the water head. The storage coefficient is given by

$$S = \rho_w \phi g \left(\beta_w + \frac{\beta_s}{\phi}\right) \tag{8}$$

where β_w is compressibility of water and β_s is the compressibility of the sediment. The water pressure can be found from

$$p_w = \rho_w(h_{w,\mathrm{IDS}} - y) \tag{9}$$

where ρ_w is the water density. The EPL is activate once the water head in the IDS reaches a the ice overburden

$$h_{w,\mathrm{IDS,max}} = \frac{\sigma_N}{\rho_w g} + y \tag{10}$$

Then, the water transfer from the IDS to the EPL is then governed by a transfer function

$$Q_t = \frac{TS}{LD}(h_{w,\mathrm{IDS}} - h_{w,\mathrm{EPL}}) \tag{11}$$

where d_{IDS} is the thickness of the IDS layer, and l is a leakage length scale. Depending on wether the transfer is from the EPL to the IDS or from the IDS to the EPL, S and D with subscript IDS and EPL are used respectively. The parameters used are given in table 1. The system is initialized with transmissivity and source flux yielding parabolic water pressure distribution which is similar to what is found in Jay-Allemand et al. [2]. A steady state system with a constant source 0.01m yr^{-1}, and the surge is triggered by an increase the source flux to 0.03m yr^{-1} lasting three months.

For the simulations we use a domain size of 25km and a minimum ice thickness of 1m to avoid zero thickness elements at the glacier front. The grid resolution is 10m in the horizontal direction and we extrude the mesh with 20 layers for the main figures and 50m and 10 layers for the systematic restarted runs for Supplementary Figure 6. The system is integrated forward in time with an adaptive forward Euler time-integration scheme.

Effective friction force with lateral friction

We add the friction force from lateral drag as an additional body force. Elmer/Ice has this feature available and it is documented in [4]. One has to supply a constant K resulting in the body force due to lateral friction. K can be (approximately) related to the glacier width $2W$ through

$$K = \frac{(n+1)^{1/n}}{W^{\frac{n+1}{n}}(2A)^{1/n}}, \tag{12}$$

under the assumption of no slip on the margins. Here, n is Glen's exponent, W is the glacier half width and A is the Arrhenius factor. For a glacier moving at high velocities, the velocity does not vary that much in the vertical direction, and we can treat the problem as one-dimensional. Then, we can approximate the lateral drag as a friction coefficient:

$$\mu_m = \frac{\tau_m}{\sigma_N} = \frac{K}{\rho g \cos(\phi)} v^{1/n}, \tag{13}$$

where ϕ is the glacier slope. The steady state friction coefficient is given by equation 2

$$\mu_{\mathrm{b,ss}} = \frac{\tau_{b,\mathrm{ss}}}{\sigma_N} = C\left(\frac{\chi}{1+\alpha\chi^q}\right)^{1/m}. \tag{14}$$

With the one-dimensional approximation of the lateral friction, the effective steady state friction force is then given by

$$\mu_{\mathrm{tot}} = \frac{\tau_{b,\mathrm{ss}} + \tau_m}{\sigma_N} = C\left(\frac{\chi}{1+\alpha\chi^q}\right)^{1/m} + \frac{K}{\rho g} v^{1/n}, \tag{15}$$

where we have approximated $\cos(\phi) \simeq 1$. From two-dimensional simulations, we can approximate the stress from the margins as a function of x as

$$\tilde{\tau}_m(x) = K \int_{y_{\mathrm{bottom}}}^{y_{\mathrm{top}}} |\vec{\mathbf{v}}(x,y)|^{1/n} \mathrm{d}y. \tag{16}$$

Linear stability

The stability of a glacier within the rate-and-state framework introduced here is not simply given by a single point reaching the velocity weakening regime. For nonzero d_c, a critical nucleation length appears from linear stability analysis in line with classical rate-and-state friction. However, in our case the nucleation length depends on the nonlinear viscous stresses rather than the shear modulus which is typically used in classical rate-and-state theory. Ruina et al. [5] performed linear stability analysis of a generalized rate-and-state friction law on the form

$$\begin{aligned} \tau_b &= F(\theta, v) \\ \dot{\theta} &= G(\theta, v). \end{aligned} \tag{17}$$

For such system, if we assume that a slider is pushed by a spring moving at a constant velocity with spring constant k, we have that the instability criterion is

$$\frac{1}{k}\left(\frac{v}{d_c}\right)\frac{\partial \tau_{\mathrm{b,ss}}}{\partial v} + 1 < 0. \tag{18}$$

The stability criterion is most useful in terms of the spring constant

$$k < k_{\mathrm{critical}} \rightarrow \text{instability} \tag{19}$$

where

$$k_{\mathrm{critical}} = -\frac{v}{d_c}\frac{\partial \tau_{\mathrm{b,ss}}}{\partial v}. \tag{20}$$

Note that for our sliding law, the instability can only occur for $\frac{\partial \tau_{\mathrm{b,ss}}}{\partial v} < 0$, i.e. in the velocity weakening regime. In addition, the spring constant has to be small enough. From k_{critical} the usual approach is to approximate a critical nucleation length from the system shear modulus. Dietrich uses the relation $k \simeq \frac{G}{L}$ [6]. However, we are dealing with ice, which is best approximated as nonlinear viscous at long time scales. Then it is more appropriate to approximate k as

$$k \simeq \frac{\dot{\epsilon}\nu^{\star}}{L} \tag{21}$$

where $\dot{\epsilon}\nu^{\star}$ is the viscous stress in a patch of size L in the velocity weakening regime. This gives a critical nucleation length

$$L_{\mathrm{critical}} \simeq -\left(\frac{v}{d_c \dot{\epsilon}\nu^{\star}}\frac{\partial \tau_{\mathrm{b,ss}}}{\partial v}\right)^{-1}. \tag{22}$$

The critical nucleation length depends on temperature through ice rheology, characteristic length scale d_c, the velocity of the sliding patch, as well as the steady state basal shear stress parameters q, A_s, σ_N m and C through the velocity derivative of $\tau_{b,ss}$ (equation 2). Although we do not explore the concept of a nucleation length in detail in this paper, it is important to bear in mind that the stability criterion for the onset of a glacier surge is not given by the sign of $\frac{\partial \tau_{\mathrm{b,ss}}}{\partial v}$ alone; a patch of size L_{critical} has to reach the velocity-weakening regime.

Scaling relation: Sliding velocity - front propagation speed

The propagation speed in a one-dimensional model is given by

$$v_{\mathrm{front}} \simeq \frac{\partial x}{\partial t} = \frac{\partial v}{\partial t} \Big/ \frac{\partial v}{\partial x} \tag{23}$$

where the derivatives are taken at the front tip. $\partial v / \partial t$ can be found from the equation relating basal shear

stress, velocity, and θ (equation 1), combined the state evolution law (equation 3).

$$\frac{\partial v}{\partial t} = \frac{\partial}{\partial t}(\frac{\tau_b}{\theta})^m A_s, \quad (24)$$

which can be rewritten as

$$\frac{\partial v}{\partial t} = -m\theta^{-m-1}\frac{\partial \theta}{\partial t} A_s \tau_b^m \quad (25)$$

At the tip, we can approximate $\tau_b \simeq \theta(\frac{v_{\text{slip}}}{A_s})^{1/m}$. This gives us the relation for the velocity derivative

$$\frac{\partial v}{\partial t} \simeq -m\theta^{-1} v_{\text{slip}} \frac{\partial \theta}{\partial t}. \quad (26)$$

We then insert for the state evolution law and find

$$\frac{\partial v}{\partial t} \simeq m\frac{v_{\text{slip}}^2}{d_c}\frac{\theta - \theta^{\dagger}(v_{\text{slip}})}{\theta}. \quad (27)$$

If the timescale of the velocity increase is short compared to the evolution of frictional strength, we can approximate $\theta \simeq 1$. If we further assume that $\theta^{\dagger}$ is small behind the surge front, we end up with the proportionality

$$\frac{\partial v}{\partial t} \sim \frac{v_{\text{slip}}^2}{d_c} \quad (28)$$

Next, assume that the velocity gradient at the front tip can be approximated by the sliding velocity at the tip multiplied by a decaying function that $f = f(x)$ that has dimension length^{-1}

$$\frac{\partial v}{\partial x} \simeq v_{\text{slip}} f(x), \quad (29)$$

The scaling relation for the front propagation speed is then

$$v_{\text{front}} \simeq \frac{\partial x}{\partial t} = \frac{\partial v_x}{\partial t} / \frac{\partial v_x}{\partial x} \sim \frac{v_{\text{slip}}}{d_c f(x)}. \quad (30)$$

Even though the function $f(x)$ is unknown, we can scale the sliding velocity with d_c in order to collapse the data.

Scaling relation: Surge front ice thickness - Front propagation speed

The thickness change can be understood from a simple conservation of mass argument. During a surge, the velocity in the vertical direction is fairly constant, so we use a one-dimensional argument. Conservation of mass for a slice at position x yields

$$y(t)\left(\frac{dv_x(x)}{dx}\right) dt = v_y(t)dt, \quad (31)$$

where we consider the propagation to occur along the x-coordinate. Assume that $dv_x(x)/dx$ is nonzero with average value to $v_{\text{slip}}/\Delta x$ in spatiotemporal interval Δx, Δt where $v_{\text{front}} = \Delta x/\Delta t$, we have that

$$\Delta y = y(\frac{\Delta x}{v_{\text{front}}}) - y(0) \simeq \int_0^{\frac{\Delta x}{v_{\text{front}}}} y(t)\frac{v_{\text{slip}}}{\Delta x} dt, \quad (32)$$

which gives

$$\Delta y \simeq y(0)\left(e^{\frac{v_{\text{slip}}}{v_{\text{front}}}} - 1\right) \quad (33)$$

This means that the elevation change in the full system is governed by $v_{\text{slip}}/v_{\text{front}}$.

Variable	Description	Value (set 1)	Value (set 2)	Unit
A	Ice rheology rate factor	15.47	158.3	$[\text{yr}^{-1}\text{MPa}^{-3}]$
A_s	Basal shear stress prefactor	100	100	$[\text{m yr}^{-1}\text{MPa}^{-3}]$
C	Peak friction coefficient	–	0.3	
C^*	Peak friction coefficient assuming $p_w \propto \sigma_n$	$\in [0.08, 0.25]$	–	
d_c	Characteristic length scale	$\in [0.1, 10]$	$\in [0.1, 1.0]$	[m]
g	Gravitational acceleration	9.81	9.81	$[\text{m s}^{-2}]$
K	Lateral friction constant	2.98×10^{-5}		$[\text{m}^{-\frac{4}{3}}\text{yr}^{\frac{1}{3}}\text{MPa}]$
m	Basal shear stress exponent	3	3	
n	Glen's exponent	3	3	
q	Sliding law postpeak exponent	$\in [1.5, 2.0]$	$\in [1.7, 2.0]$	
SMB	Surface mass balance			$[\text{m yr}^{-1}]$
v_b	Basal velocity			$[\text{m yr}^{-1}]$
v_s	Surface velocity			$[\text{m yr}^{-1}]$
v_{slip}	Sliding velocity (100m behind surge front)			$[\text{m yr}^{-1}]$
v_{front}	Surge front propagation velocity			$[\text{m yr}^{-1}]$
y_0	Initial glacier thickness			[m]
Δy	Change in glacier thickness			[m]
θ	State parameter (Degree of cavitation)			
$\theta^\dagger$	Steady state θ			
ρ	Density	915	915	$[\text{kg m}^{-3}]$
σ_n	Normal stress			[Pa]
σ_N	Effective normal stress			[Pa]
τ_b	Basal shear stress			[Pa]
$\tilde{\tau}_m$	Shear stress from margins			[Pa]
τ_{tot}	Driving stress			[Pa]
$\mu_{b,ss}$	Steady state basal friction coefficient			
μ_m	Approximate friction coefficient from margins			
μ_{tot}	Total friction coefficient			
T_{IDS}	IDS transmissivity	–	$\frac{10^{-2}x[\text{m}]}{\frac{6(x[\text{m}]-10000)}{10^6}+\frac{20}{67}e^{-x[\text{m}]/6700}} + 1.0$	$[\text{m yr}^{-2}]$
T_{EPL}	EPL transmissivity	–	2.5×10^5	$[\text{m yr}^{-2}]$
Q	Source flux	–	$\in [0.01, 0.03]$	$[\text{m yr}^{-1}]$
S	Storage coefficient	–		$[\text{m}^{-1}]$
h_w	Water head	–		[m]
ρ_w	Water density	–	1000	$[\text{kg m}^{-3}]$
β_w	Water compressibility	–	5.04×10^{-4}	$[\text{MPa}^{-1}]$
β_s	Sediment compressibility	–	10^{-2}	$[\text{MPa}^{-1}]$
ϕ_{IDS}	IDS porosity	–	0.4	
ϕ_{EPL}	EPL porosity	–	0.8	
D_{IDS}	IDS thickness	–	1.0	[m]
D_{EPL}	EPL thickness	–	5.0	[m]
L	Leakage length scale	–	2.0	[km]

Supplementary Table 1: List of variables. Set 1 assumes proportionality between water pressure and normal stress, while set 2 uses a full hydrology model.

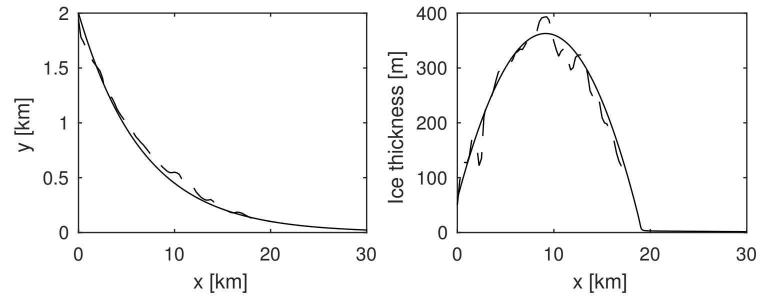

Supplementary Figure 1: Initial glacier geometry used in the simulations in this paper. To model a realistic glacier geometry, we parametrize the Variegated glacier geometry data from 1973 [2].The bottom geometry follows $y = 2000\text{m}e^{-x/6700\text{m}}$ while the thickness follows $y = 61.5\text{m} + 0.066x - 3.6 \times 10^{-6}x^2$ with a smoothed step function to avoid negative thickness at the front.

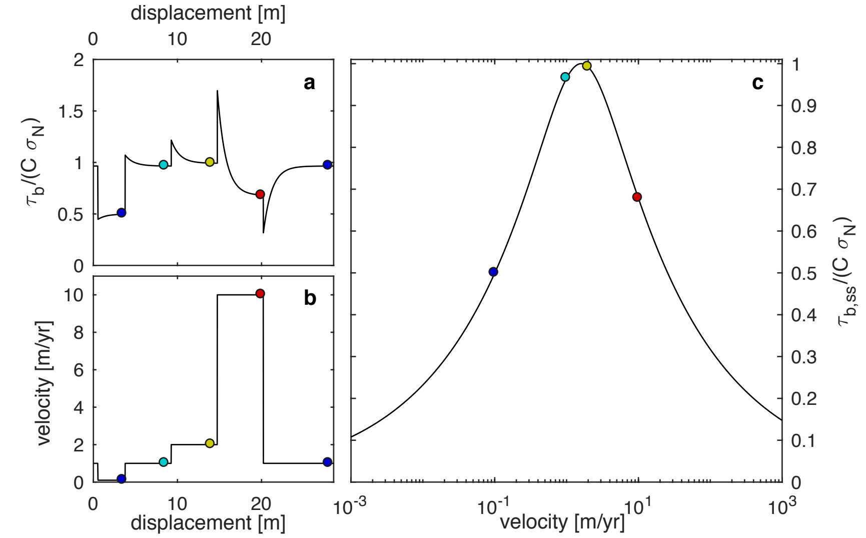

Supplementary Figure 2: Qualitative features of the sliding law introduced in this paper when used on a rigid slider with enforced velocity. The slider subject to step changes in velocity of $[1, 0.1, 2, 10, 1]$m/yr (b). A change in velocity results in a direct effect according to equation 1 (a). Then the friction coefficient approaches the steady state value (c) according to the state evolution law. Qualitatively, this response matches recent experimental observations (not shown) [7, 8]. Parameters used: $A_s = 100\text{m MPa}^{-3}\text{yr}^{-1}$, $d_c = 1\text{m}$, $\sigma_N = 1\text{MPa}$, $C = 0.2$, $n = 3$, $m = 3$, $q = 2.0$.

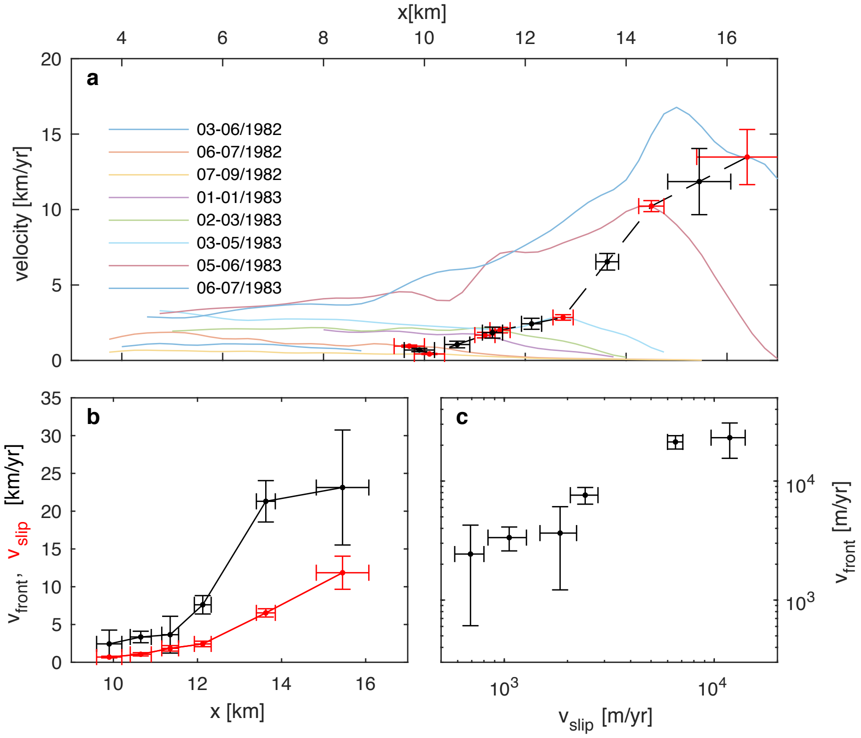

Supplementary Figure 3: (a): Flow velocities during the Variegated glacier surge from Jay-Allemand et al. [2]. The red crosses marks the front tip where the errorbars are based on our confidence in determining the surge front position and velocity. We have evaluated the surge speed in as the difference between the front positions and used linear interpolation for the sliding speed in the center of the interval (black crosses). (b): The front and sliding speed as a function of x. (c): Relation between front speed and sliding speed. The data from this figure is used to test the scaling relation between sliding speed and front speed in the main text.

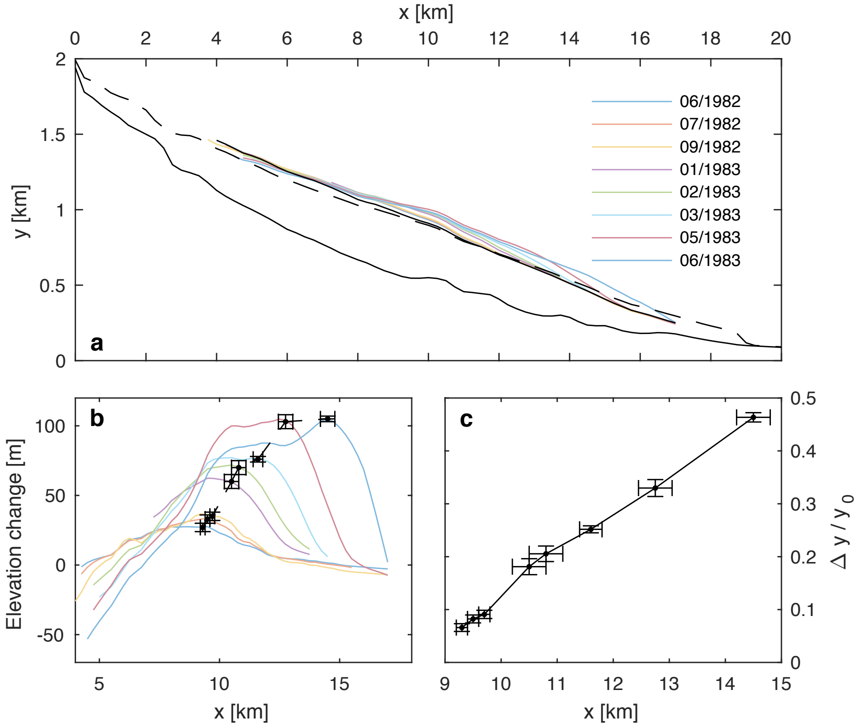

Supplementary Figure 4: (a): Evolution of glacier topography during the Variegated glacier surge from Jay-Allemand et al. [2]. The black lines show the geometry from 09/1981 used to determine y_0. The dashed black line show the geometry from 1973 for reference. (b): Change in elevation from the initial geometry. The black crosses show the thickness at the surge front where the errorbars that are used show our confidence in determining the position and elevation change. (c): Relative elevation change $\Delta y/y_0$ as a function of x, where we use the profile from The data from this figure is used to test the scaling relation between relative elevation change, sliding speed and front speed in the main text.

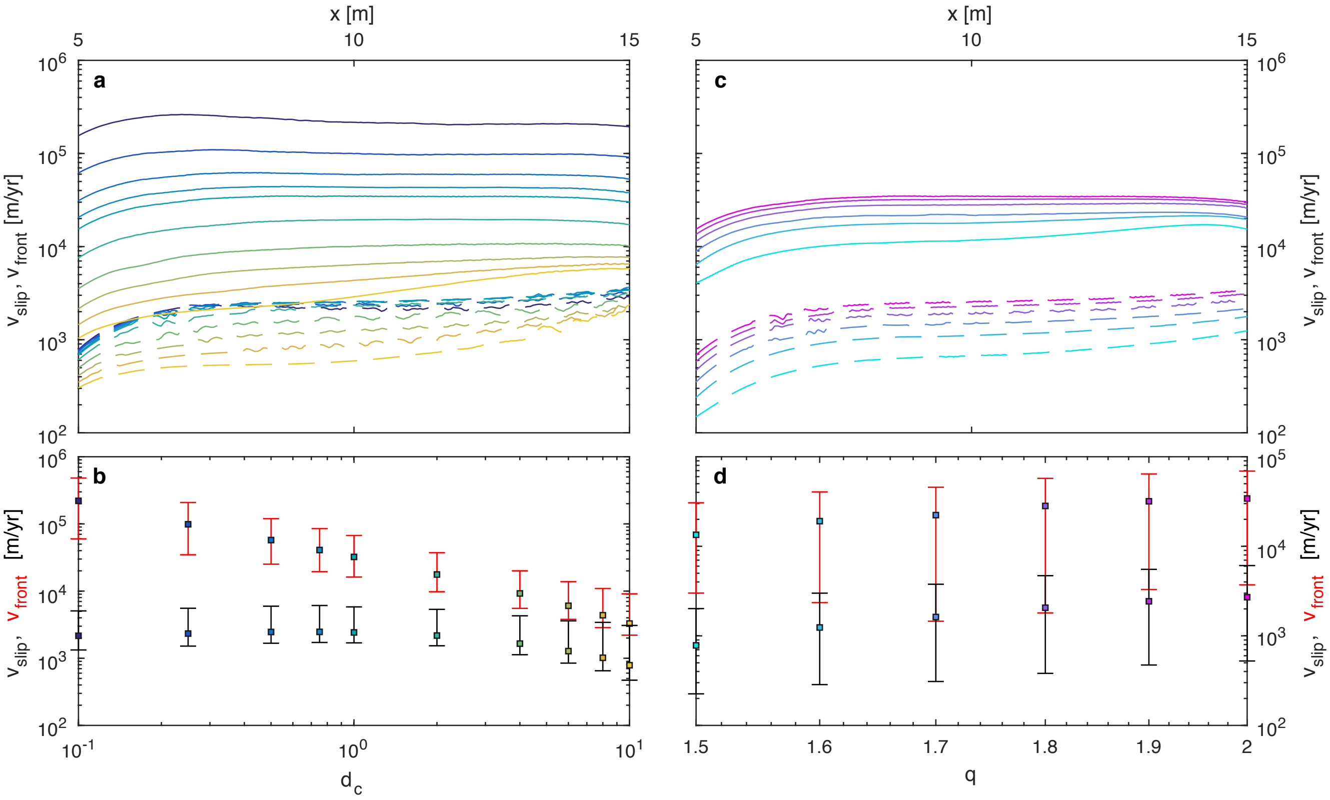

Supplementary Figure 5: Sensitivity of front propagation to changes in d_c and q. (a): The front propagation speed v_{front} (solid lines) and sliding speed v_{slip} (dashed lines) as a function of x for different values of d_c for $q = 2.0$ with colors corresponding to (b). In (b), v_{front} and v_{slip} are plotted as functions of d_c. The errorbars show the minimum and maximum values. (c): The front propagation speed v_{front} (solid lines) and sliding speed v_{slip} (dashed lines) as a function of x for different values of q for $d_c = 1.0$m with colors corresponding to (d). In (d), v_{front} and v_{slip} are plotted as functions of q. The errorbars show the minimum and maximum values.

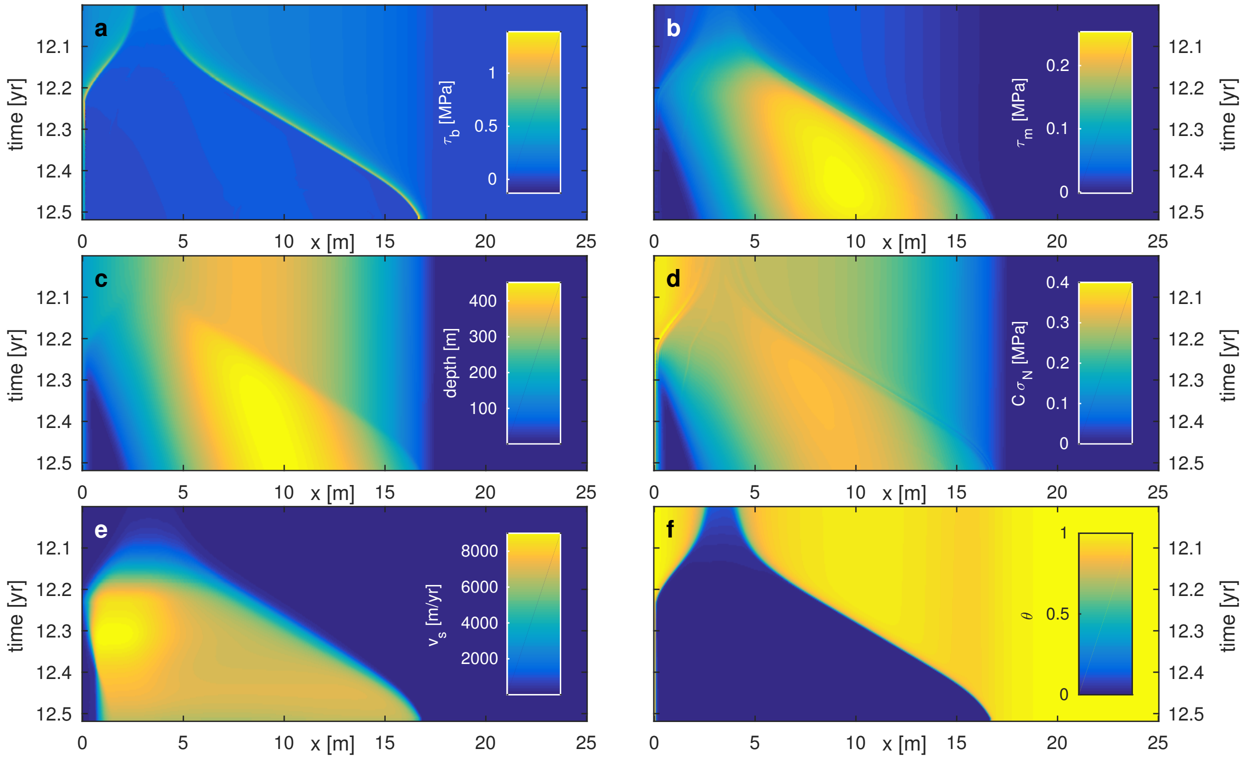

Supplementary Figure 6: Spatiotemporal evolution of basal variables during a surge with $d_c = 1.0$m and $q = 2.0$. (a): Basal shear stress. (b): Depth. (c): Surface velocity. (d): Stress from margins. (e): Effective normal stress. (f): State parameter.

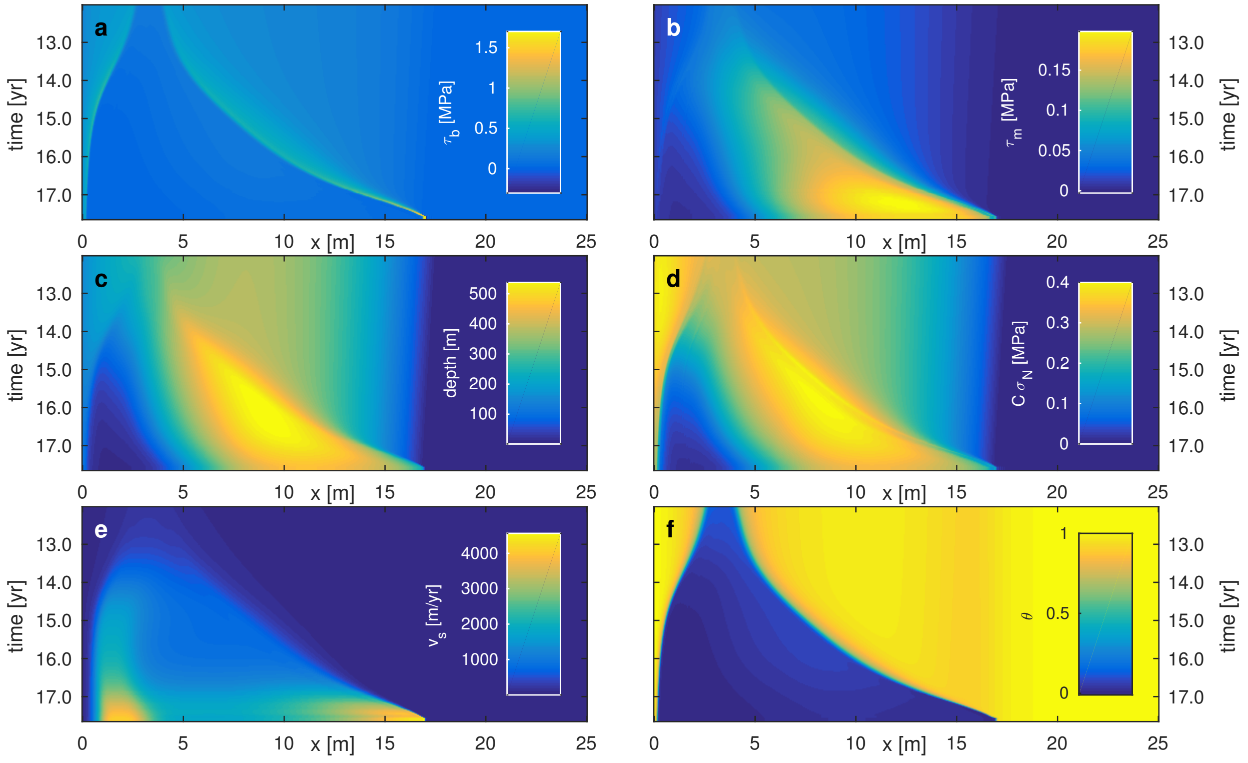

Supplementary Figure 7: Spatiotemporal evolution of basal variables during a surge with $d_c = 10.0$m and $q = 2.0$. (a): Basal shear stress. (b): Depth. (c): Surface velocity. (d): Stress from margins. (e): Effective normal stress. (f): State parameter.

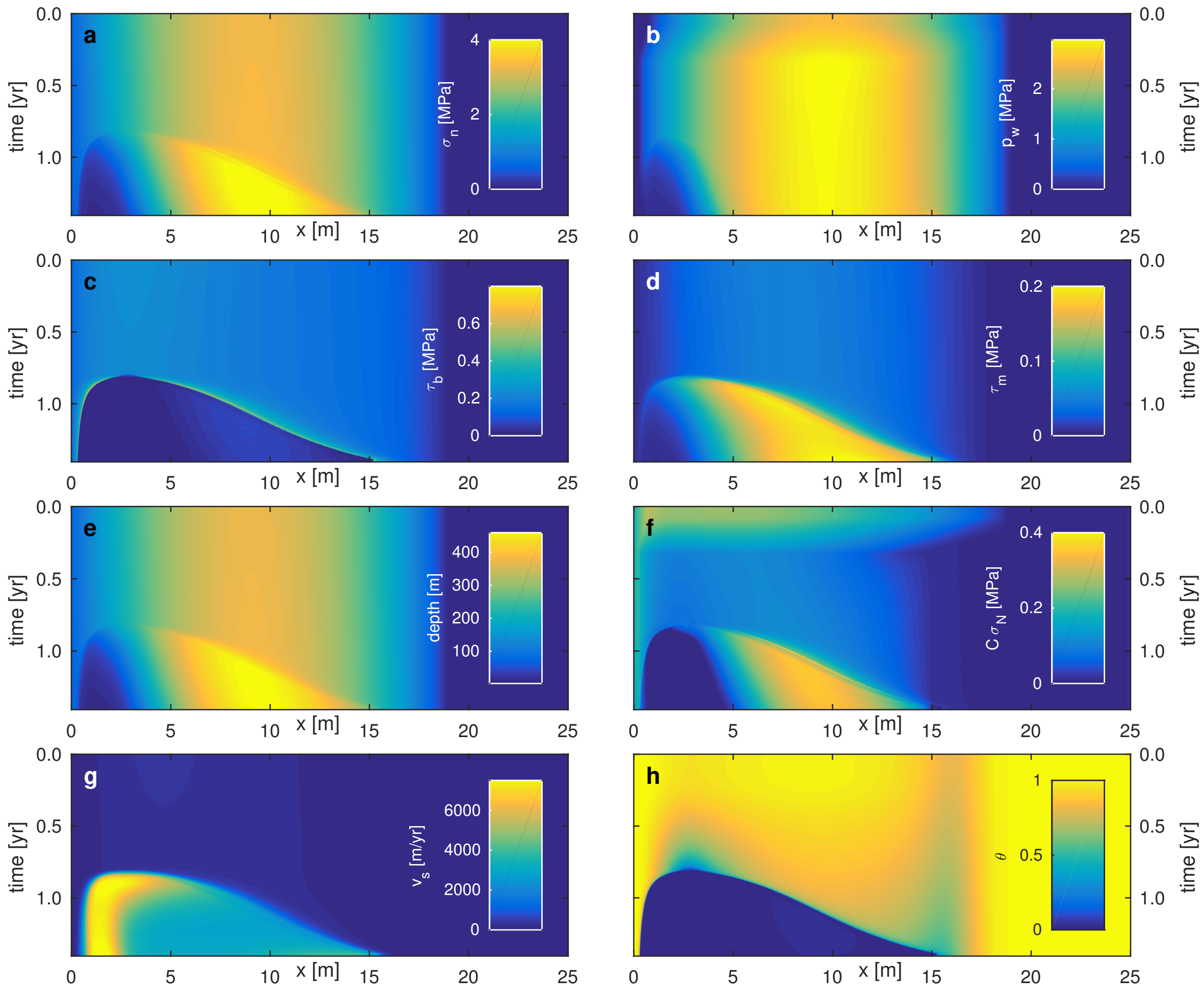

Supplementary Figure 8: Spatiotemporal evolution of basal variables during a surge using the full hydrology model with $d_c = 1.0$m and $q = 2.0$. (a): Normal stress. (b): Water pressure. (c): Basal shear stress. (d): Depth. (e): Surface velocity. (f): Stress from margins. (g): Effective normal stress. (h): State parameter.

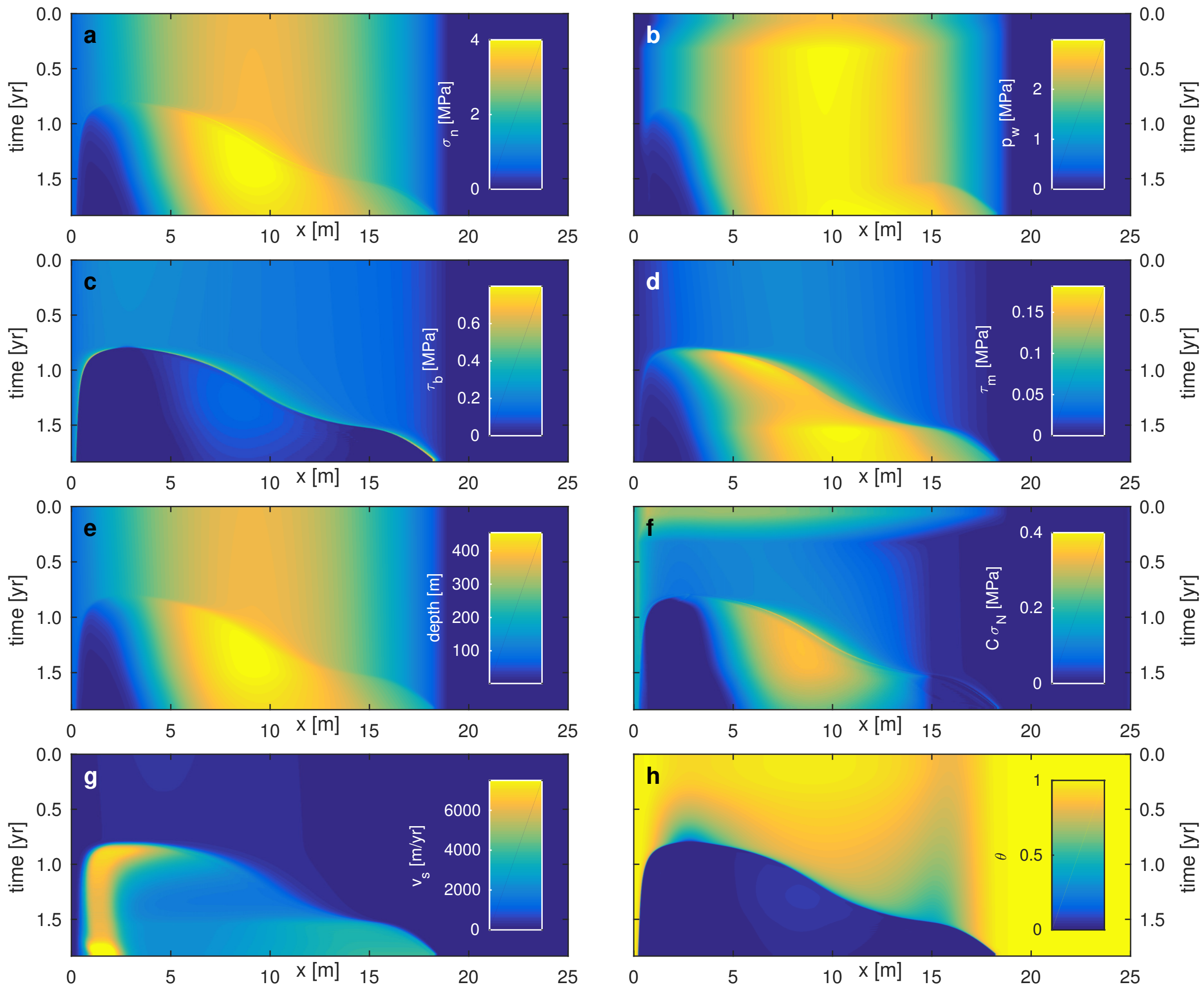

Supplementary Figure 9: Spatiotemporal evolution of basal variables during a surge using the full hydrology model with $d_c = 1.0$m and $q = 1.7$. (a): Normal stress. (b): Water pressure. (c): Basal shear stress. (d): Depth. (e): Surface velocity. (f): Stress from margins. (g): Effective normal stress. (h): State parameter.

[1] Gagliardini, O. *et al.* Capabilities and performance of elmer/ice, a new-generation ice sheet model. *Geoscientific Model Development* **6**, 1299–1318 (2013).

[2] Jay-Allemand, M., Gillet-Chaulet, F., Gagliardini, O. & Nodet, M. Investigating changes in basal conditions of variegated glacier prior to and during its 1982-1983 surge. *The Cryosphere* **5**, 659–672 (2011).

[3] Fleurian, B. d. *et al.* A double continuum hydrological model for glacier applications. *The Cryosphere* **8**, 137–153 (2014).

[4] Gagliardini, O., Durand, G., Zwinger, T., Hindmarsh, R. & Le Meur, E. Coupling of ice-shelf melting and buttressing is a key process in ice-sheets dynamics. *Geophysical Research Letters* **37** (2010).

[5] Ruina, A. Slip instability and state variable friction laws. *Journal of Geophysical Research: Solid Earth* **88**, 10359–10370 (1983).

[6] Dieterich, J. H. Modeling of rock friction: 1. experimental results and constitutive equations. *Journal of Geophysical Research: Solid Earth* **84**, 2161–2168 (1979).

[7] Zoet, L. *et al.* The effects of entrained debris on the basal sliding stability of a glacier. *Journal of Geophysical Research: Earth Surface* **118**, 656–666 (2013).

[8] McCarthy, C., Savage, H. & Nettles, M. Temperature dependence of ice-on-rock friction at realistic glacier conditions. *Phil. Trans. R. Soc. A* **375**, 20150348 (2017).
